# Giant Cell Arteritis as a Form of Pembrolizumab-Associated Vascular Toxicity

**DOI:** 10.1016/j.jaccas.2026.107742

**Published:** 2026-04-01

**Authors:** Daichi Umemoto, Hiroyuki Nagano, Keiichiro Kinoshita, Shunsuke Tagawa, Hirofumi Miyake, Shinji Sumiyoshi, Kazuhiro Hatta, Eri Iwai-Kanai

**Affiliations:** a Department of General Internal Medicine, Tenri Hospital, Tenri, Japan; b Department of Diagnostic Pathology, Tenri Hospital, Tenri, Japan; c Faculty of Health Care, Tenri University, Tenri, Japan

**Keywords:** giant cell arteritis, immune checkpoint inhibitor, new-onset headache, pembrolizumab, vasculitis

## Abstract

**Background:**

As the use of immune checkpoint inhibitors becomes more prevalent in cancer treatment, physicians should be aware of immune-related adverse events. Cardiovascular toxicity is of particular concern because of the risk of serious consequences, yet little is known about these immune-related adverse events including giant cell arteritis (GCA).

**Case Summary:**

A 68-year-old man complained of new-onset daily persistent headache and fever after pembrolizumab initiation for renal cell carcinoma. The patient was negative for vasculitis-associated autoantibodies and showed no significant findings on ultrasound or contrast-enhanced computed tomography. However, contrast-enhanced magnetic resonance imaging revealed gadolinium enhancement in both temporal arteries, and temporal artery biopsy showed findings consistent with GCA. Prednisolone was initiated immediately and dramatically improved the clinical symptoms, which prevented vision loss.

**Conclusions:**

This case highlights the importance of considering GCA as a possible diagnosis in patients receiving immune checkpoint inhibitors therapy who report development of new headache.

## Visual Summary

**Figure:**
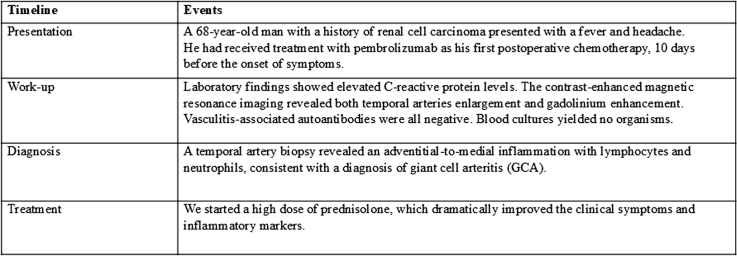

Immune checkpoint inhibitors (ICIs) have become a major oncological therapy and have improved cancer outcomes. However, they frequently cause immune-related adverse events (irAEs), affecting over half of treated patients.^1^ Pembrolizumab is an ICI that targets programmed cell death receptor 1 and has been shown to be effective against various solid tumors, including melanoma, non-small-cell lung cancer, and renal cell carcinoma. As the indications of this drug has rapidly increased, physicians are likely to encounter patients with pembrolizumab-associated irAEs.

Although cardiovascular toxicity is rare compared to dermatologic, gastrointestinal, or endocrine toxicities, it is one of the most important irAEs because of its high fatality rate.^2^^,^^3^ The most commonly reported cardiovascular irAE is myocarditis, with an incidence rate ranging from 0.75% to 2%. Other cardiovascular irAEs include pericarditis, conduction disorders, heart failure, and pericardial effusions, with prevalences of 0.55%, 0.85%, 3.4%, and 3.98%, respectively.^4^ ICIs-related vasculitis has also been reported, but the estimated risk is not well-defined owing to the limited available data. A retrospective multicenter European study reported on 27 adult patients with ICI-related large-vessel vasculitis between March 2018 and August 2024.^1^ Among ICI recipients, giant cell arteritis (GCA) was reported in only 0.05% of cases, but accurate incidence rates are difficult to calculate given its rarity. GCA is the most common inflammatory rheumatic large-vessel vasculitis in adults and causes severe headaches, scalp tenderness, jaw pain, and potential vision loss. Urgent treatment with corticosteroids is essential for preventing blindness, which is a medical emergency.

Herein, we present the case of a patient who was treated with pembrolizumab as his first postoperative chemotherapy for renal cell carcinoma and subsequently developed a new-onset, daily, persistent headache. He was diagnosed with pembrolizumab-related GCA and was administered high-dose corticosteroids immediately, resulting in a rapid improvement of clinical symptoms. In this case, early consideration of the possibility of GCA and careful examination of imaging features helped avoid vision loss, which is a serious medical condition.

## History of Presentation

A 68-year-old man was admitted to our hospital owing to fever and headache. Two months previously, he underwent nephrectomy for renal cell carcinoma. The postoperative course was uneventful, and he received pembrolizumab as his first postoperative chemotherapy 10 days before the onset of symptoms. He had a history of chronic kidney disease and hyperuricemia but did not take any medication.

Physical examination revealed a temperature of 38.4 °C, and bilateral temporal arteries were palpable, tender, and thickened. The patient experienced jaw claudication but had no visual symptoms. Other than a persistent headache, he did not complain of any pain. This included the bilateral shoulder and hip girdle pain that was characteristic of polymyalgia rheumatica. Laboratory findings showed an elevated white blood cell count of 8,860/μL, elevated C-reactive protein levels of 22.5 mg/dL, and a markedly elevated erythrocyte sedimentation rate of over 100 mm/h. All cardiac enzymes, including creatine kinase, lactate dehydrogenase, and troponin, are within the normal range. All vasculitis-associated autoantibodies tested negative (Table 1). Blood cultures did not yield any organisms.

**Table 1:** Laboratory Findings

Hematology		Biochemistry			
WBC	8,860 /μL	TP	7.2 g/dL	RF	<5.0 IU/mL
Neut	81 %	Alb	2.6 g/dL	IgG	1932 mg/dL
Lym	8.0 %	AST	31 U/L	IgA	267 mg/dL
Mono	7.0 %	ALT	34 U/L	IgM	31 mg/dL
Eos	3.0 %	T-Bil	0.7 mg/dL	C3	186.5 mg/dL
Baso	1.0 %	γ-GTP	134 U/L	C4	33.4 mg/dL
RBC	3.42×10^6^/μL	ALP	235 U/L	Ferritin	2,648 ng/mL
Hb	10.0 g/dL	CK	24 U/L	ANA	<40×
Plt	38.7×10^4^/μL	BUN	13.7 mg/dL	dsDNA Ab	<10 IU/mL
		Cre	1.39 mg/dL	Sm Ab	<1.0 U/mL
		Na	138 mEq/L	SS-A Ab	<1.0 U/mL
		K	4.3 mEq/L	SS-B Ab	<1.0 U/mL
		Cl	101 mEq/L	MPO-ANCA	<1.0 IU/mL
		CRP	22.5 mg/dL	PR3-ANCA	<1.0 IU/mL
		ESR	>100 mm/h	LDH	212 U/L
		TnT	<0.01 ng/mL		

Alb = albumin; ALP = alkaline phosphatase; ALT = alanine aminotransferase; ANA = antinuclear antibody; AST = aspartate aminotransferase; Baso = basophil; BUN = blood urea nitrogen; CK = creatine kinase; Cl = chloride; Cre = creatinine; CRP = C-reactive protein; dsDNA Ab = double-stranded DNA antibody; Eos = eosinophil; ESR = erythrocyte sedimentation rate; Hb = hemoglobin; K = potassium; LDH = lactate dehydrogenase; Lymph = lymphocyte; Mono = monocyte; MPO-ANCA = myeloperoxidase–ANCA; Na = sodium; Neut = neutrophil; Plt = platelet; PR3-ANCA = proteinase 3–ANCA; RBC = red blood cell; RF = rheumatoid factor; Sm Ab = Smith antibody; SS-A = anti–SS-A (Ro) antibody; SS-B = anti–SS-B (La) antibody; T-Bil = total bilirubin; TnT = Troponin T; TP = total protein; WBC = white blood cell; γ-GTP = gamma-glutamyl transpeptidase.

**Figure 1:**
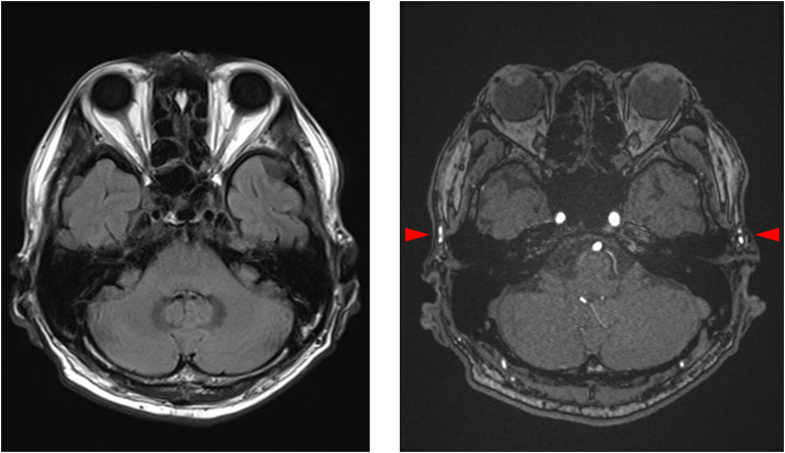
Contrast-Enhanced MRI of the Brain A contrast-enhanced magnetic resonance imaging (MRI) of the brain showed that both temporal arteries were slightly enlarged and displayed some gadolinium enhancement (red arrows).

**Figure 2:**
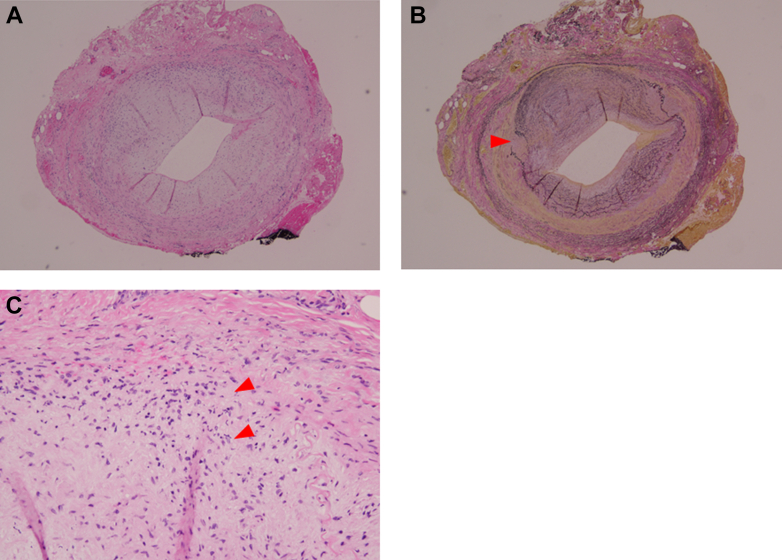
Microscopic View of a Temporal Arterial Specimen The histological findings of the temporal artery revealed adventitial-to-medial inflammation with lymphocytes and neutrophils (red arrow in C), as well as disruption to the elastic lamina (red arrows in B). (A) Hematoxylin-eosin, objective (lens) 4×. (B) Elastica Van Gieson, objective (lens) 4×. (C) Hematoxylin-eosin, objective (lens) 20×.

Ultrasonography and contrast-enhanced computed tomography of the chest and trunk showed no significant thickening of the large vessel walls. Contrast-enhanced magnetic resonance imaging of the brain also showed no thickening of the large vessel walls but revealed gadolinium enhancement of the bilateral temporal artery walls (Figure 1). Therefore, we urgently performed a temporal artery biopsy, which revealed adventitial-to-medial inflammation with lymphocytes and neutrophils, as well as disruption of the lamina (Figure 2). These findings were consistent with the diagnosis of GCA.

**Figure 3:**
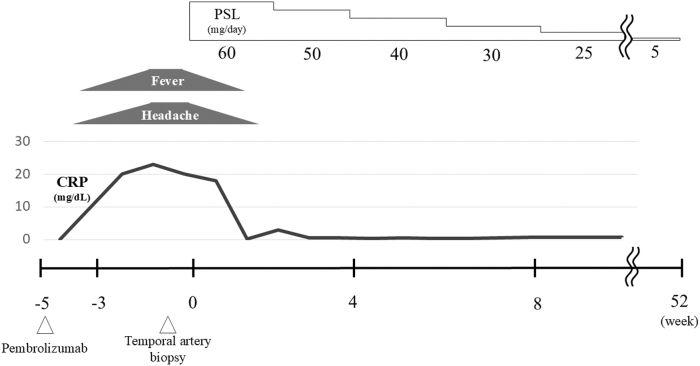
The Patient's Clinical Course CRP = C-reactive protein; PSL = prednisolone.

We immediately initiated high-dose steroid therapy with 60 mg (1 mg/kg/d) of prednisolone on day 14, which resulted in a dramatic improvement of clinical symptoms and inflammation markers. The patient transitioned to an oral prednisone taper and was discharged on day 23. Pembrolizumab treatment was discontinued. One year after treatment initiation, the patient remained asymptomatic and received outpatient follow-up care for oral glucocorticoid therapy (Figure 3). Cancer management was well-controlled, and no recurrence was observed.

## Discussion

Our case demonstrates that GCA can occur as an irAE in patients undergoing pembrolizumab treatment and that prompt diagnosis and therapeutic intervention can prevent serious complications. Inhibiting the programmed cell death receptor 1 checkpoint has been reported to lead to T-cell-mediated inflammation and arterial wall damage, which can accelerate the autoimmune process in GCA.^5^ A recent systematic literature review indicated that ICI-GCAs have a prevalence of 0.06% among ICI recipients.^6^ Despite its rarity, its clinical manifestations can be serious and include permanent vision loss.^7^^,^^8^ Visual impairment is also a significant complication of primary GCA. However, the reported incidence of vision loss is much higher in ICI-related GCA than in primary GCA (<20%).^9^ A delayed diagnosis and initiation of corticosteroids in cases of ICI-related GCA may result in more severe outcomes. In our case, a careful examination of the brain magnetic resonance imaging scan (Figure 1) resulted in a subsequent histological examination of the temporal arteries (Figure 2). This enabled us to make a diagnosis and provide therapeutic intervention without delay.

Our case is also clinically significant because glucocorticoids equivalent to 1 mg/kg/d prednisolone, a therapeutic dose similar to that used for primary GCA, were effective. Standard management of primary GCA includes high-dose glucocorticoids with or without immunosuppressants. However, these therapies may attenuate the anticancer effects of ICIs and potentially worsen patient outcomes. Healthcare professionals across all medical fields are required to ensure the best possible management of ICI-related adverse events in these patients.

The European retrospective multicenter study indicates that the median duration of ICIs therapy preceding symptom onset was 3 months.^1^ However, case reports have shown a wide range of onset times, from symptom development within weeks of administration to more than a few years later.^10^

In our case, the patient experienced a headache and fever 10 days after receiving pembrolizumab. C-reactive protein levels were also elevated at the same time despite being normal on the day the drug was administered (Figure 3). To date, the clinical features have not been well defined owing to the limited amount of available data. However, pembrolizumab is becoming increasingly widely used. Physicians, including cardiovascular specialists and general internists, must be aware of the risk of vasculitis in patients undergoing ICIs therapy regardless of the timing of symptom onset. Further prospective studies are needed to provide more information on these topics.

## Conclusions

Herein, we present a case of a patient who developed GCA as a possible irAE 10 days after the initiation of pembrolizumab for renal cell carcinoma. Given the increasing use of ICIs in patients with cancer, physicians should be aware of the potential for vascular emergencies to facilitate prompt recognition and treatment, particularly in patients reporting new headaches or jaw claudication despite their rarity.

**Visual Summary:**
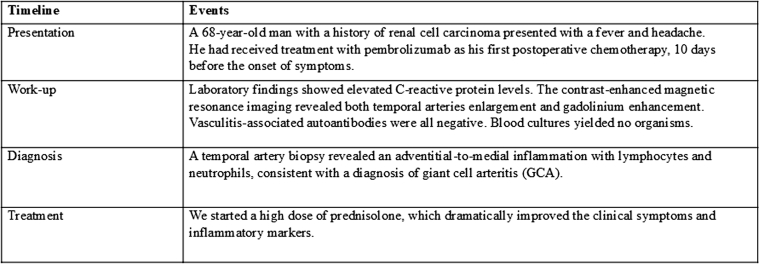
Timeline of the Case

## Funding Support and Author Disclosures

This work was supported in part by grants to Dr Iwai-Kanai from the Ministry of Education, Science, and Culture of Japan. The authors have reported that they have no relationships relevant to the contents of this paper to disclose.

**Take-Home Message**

• Although giant cell arteritis is an extremely rare immune-related adverse event, it is important to include giant cell arteritis in the differential diagnosis of patients receiving immune checkpoint inhibitors therapy who report new headaches or jaw claudication to avoid the development of serious complications such as vision loss.
